# Supplementary material for: Efficacy of Nanofiber Sheets Incorporating Lenvatinib in a Hepatocellular Carcinoma Xenograft Model
Source: Nanomaterials (Basel). 2022 Apr 15;12(8):1364. doi: 10.3390/nano12081364 (PMC9025678; doi:10.3390/nano12081364)
Supplement: Supplementary file 1 [file nanomaterials-12-01364-s001.zip › nanomaterials-1630095-supplementary.pdf]

Supplementary Materials

Efficacy of Nanofiber Sheets Incorporating Lenvatinib in a Hepatocellular Carcinoma Xenograft Model

Terufumi Yoshida [1], Masaki Kaibori [1,*], Nanami Fujisawa [2], Mariko Ishizuka [1], Fusao Sumiyama [1], Masahiko Hatta [1], Hisashi Kosaka [1], Kosuke Matsui [1], Kensuke Suzuki [3], Tomoya O. Akama [4], Tayo Katano [5], Kengo Yoshii [6], Mitsuhiro Ebara [2] and Mitsugu Sekimoto [1]

1 Department of Surgery, Kansai Medical University, 2-5-1 Shinmachi, Hirakata 573-1010, Japan; yoshiter@hirakata.kmu.ac.jp (T.Y.); ishizukm@takii.kmu.ac.jp (M.I.); sumiyamf@hirakata.kmu.ac.jp (F.S.); hattamas@hirakata.kmu.ac.jp (M.H.); kosakahi@hirakata.kmu.ac.jp (H.K.); matsuik@hirakata.kmu.ac.jp (K.M.); sekimotm@hirakata.kmu.ac.jp (M.S.)
2 Research Center for Functional Materials, National Institute for Materials Science (NIMS), Tsukuba 305-0044, Japan; nfujisawa.tokyo@gmail.com (N.F.); ebara.mitsuhiro@nims.go.jp (M.E.)
3 Department of Otolaryngology, Head and Neck Surgery, Kansai Medical University, Hirakata 573-1010, Japan; suzukken@hirakata.kmu.ac.jp
4 Department of Pharmacology, Kansai Medical University, Hirakata 573-1010, Japan; akamat@hirakata.kmu.ac.jp
5 Department of Medical Chemistry, Kansai Medical University, Hirakata 573-1010, Japan; katanot@hirakata.kmu.ac.jp
6 Department of Mathematics and Statistics in Medical Sciences, Kyoto Prefectural University of Medicine, Kyoto 606-0823, Japan; yoshii-k@koto.kpu-m.ac.jp
* Correspondence: kaibori@hirakata.kmu.ac.jp; Tel.: +81-72-804-0101 (ext. 56130)

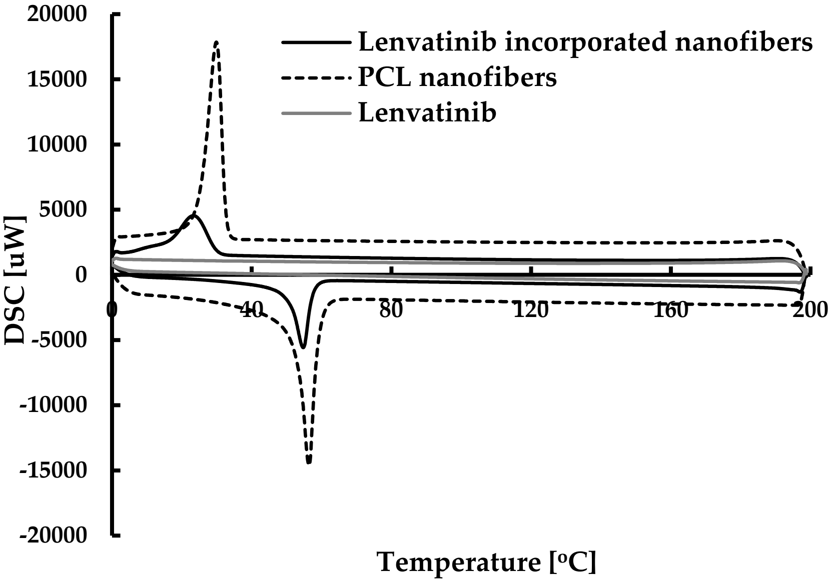

Figure S1: DSC curves of lenvatinib incorporated nanofibers, PCL nanofibers and lenvatinib.
